# High levels of depressive symptoms and low quality of life are reported during pregnancy in Cape Coast, Ghana; a longitudinal study

**DOI:** 10.1186/s12889-022-13299-2

**Published:** 2022-05-05

**Authors:** Ruth Adisetu Pobee, Jacob Setorglo, Moses Kwashie Klevor, Laura E. Murray-Kolb

**Affiliations:** 1 Department of Nutritional Sciences, The Pennsylvania State University, University Park, PA 16802 USA; 2 Department of Clinical Nutrition and Dietetics, University of Cape Coast, Cape Coast, Ghana; 3 Department of Nutrition Science, Purdue University, Room 214 Stone Hall, 700 West State Street, West Lafayette, IN 47907 USA

**Keywords:** Depression, Anxiety, Quality of life, Pregnancy, Ghana

## Abstract

**Background:**

Significant rates of anxiety, depressive symptoms, and low quality of life (QoL) have been found among pregnant women in developed countries. These psychosocial disturbances have not been adequately assessed during pregnancy in many developing countries.

**Methods:**

Women were recruited in their first trimester of pregnancy (< 13 weeks; *n* = 116) and followed through to their 2^nd^ (*n* = 71) and 3^rd^ (*n* = 71) trimesters. Questionnaires were used to collect data on anxiety symptoms (Beck Anxiety Inventory; BAI), depressive symptoms (Center for Epidemiological Studies-Depression Inventory; CES-D), and quality of life (RAND SF-36; QoL). Psychometric analyses were used to determine the reliability of the questionnaires in this context. The proportion of pregnant women with psychosocial disturbances at each trimester was determined. Repeated measures ANOVA were used to examine changes in psychosocial outcomes over time; and generalized estimating equation to determine if gestational age predicted the psychosocial outcomes whilst controlling for sociodemographic variables.

**Results:**

Participants were aged 27.1 ± 5.2 years, on average. Psychometric analyses revealed a 4-factor solution for BAI (18 items), 1-factor solution for CES-D (13 items) and 4-factor solution for RAND SF-36 (26 items). The prevalence estimate of psychosocial disturbances was 34%, 10%, 2% (anxiety), 49%, 31%, 34% (depressive symptoms), and 46%, 37%, 59% (low QoL) for 1^st^, 2^nd^ and 3^rd^ trimesters, respectively. Gestational age and food insecurity were significant predictors of depressive symptoms, anxiety symptoms and QoL.

**Conclusions:**

In this population of Ghanaian women, the levels of depressive symptoms and low QoL observed across pregnancy should be recognized as major public health problems and efforts to address these should be put in place. Addressing food insecurity may be a major step to solve not only the physical needs of the pregnant woman but also the psychological needs.

**Supplementary Information:**

The online version contains supplementary material available at 10.1186/s12889-022-13299-2.

## Background

In many developed countries, depressive symptoms are assessed during pregnancy [1, 2]. This is not the case in many developing countries, including Ghana where depression is considered a myth, linked to psychosis, and is thus stigmatized [3]. In fact, most Ghanaian languages have no name for it [4]. However, depression and anxiety commonly occur during pregnancy and often coincide with the greater demands in advancing pregnancy and the hormonal changes that occur [5–7]. According to recent reports, antenatal depressive symptoms affect up to 29% of pregnant women and may vary by trimester [8–15]. Prevalence rates of 7.4%, 12.8% and 12% for depression, and 13%, 4% and 6% for anxiety, have been reported in the 1^st^, 2^nd^ and 3^rd^ trimesters respectively [16, 17]. Furthermore, antenatal depression has been associated with lower gestational age at birth (< 37 weeks) and poor pregnancy outcomes [18]. Other predictors of depressive or anxiety symptoms include low levels of education, multiparity, history of depression, severe nausea, extreme fatigue, lack of physical exercise and sleep, negative life events, and food insecurity [19–23]. Not living with a partner and having an unplanned pregnancy or a long time to pregnancy have been associated with depressive and/or anxiety symptoms in early pregnancy only [23]. Maternal depression and anxiety can have devastating consequences for the mother and fetus*.* Anxiety during pregnancy can lead to shorter gestation and adverse effects on fetal neurodevelopment and child outcomes [24, 25]. Both anxiety and depression in mothers during pregnancy are associated with preterm delivery and low birth weight infants [25]. Depression may also impact the woman’s biological and social functioning [26, 27] and lead to self-harm and suicide ideation [28]. This may further lead to a reduced quality of life (QoL) during and after pregnancy [29]. Despite this, anxiety and depression remain underdiagnosed and undertreated during pregnancy, especially in developing countries [17]. A major limitation of most studies that have examined depressive symptoms during pregnancy is their cross sectional nature where causal or relational inference cannot be made [16, 30, 31]. Longitudinal studies assessing QoL during pregnancy mostly examined differences in mean scores across trimesters [29, 32–34], compared QoL in healthy and non-healthy pregnancies [35–38], or looked at trajectory patterns in QoL across trimesters [39, 40]. Additionally, few studies have examined the relation between gestational age and psychosocial outcomes.

In Ghana, few studies have focused on depression and anxiety during pregnancy [41, 42], and we only found one study that examined QoL among pregnant women [43]. This is true despite the fact that a high prevalence of these psychosocial disturbances may exist [44]. There is a need for longitudinal studies that assess these psychosocial outcomes to estimate prevalence throughout pregnancy so that interventions targeted at curbing their deleterious effects can be developed and tested. In Ghana, anxiety and depression are particularly neglected and there are numerous reasons for this neglect, including cultural beliefs and attitudes, low priority given to mental health, inadequate mental health facilities, and insufficient routine data collection [44]. As such, the magnitude of the problem is neither understood nor diagnosed. Furthermore, other sociocultural norms and values about pregnancy and childbearing exist in Ghana. Most ethnic groups in Ghana are pronatalist, which makes childbirth an index of both femininity and masculinity. Women usually come under intense pressure to perpetuate and sustain the family lineage. Thus, whereas pregnancy is highly valued, elevated anxiety could arise due to fears of losing the pregnancy. The cultural norms, beliefs, attitudes towards mental health, the lack of priority, and mental health facilities may influence not only help-seeking behaviors but stigma, caregiving and social inclusion.

In order to assess depression, anxiety and QoL in pregnant women from Ghana, the reliability of the instruments used to assess these psychosocial outcomes needs to be determined because most of the instruments have been developed in Western countries and the reliability is not yet known for many developing countries. As such, it is important to test the reliability of the questionnaires, particularly in the pregnant Ghanaian setting, before using them to interpret results.

Our aim was to conduct psychometric analyses to determine the reliability of the questionnaires used to assess psychosocial outcomes, then establish the prevalence estimate of depressive and anxiety symptoms and low QoL throughout pregnancy in Cape Coast, Ghana. Given prior findings in other regions of the world, we hypothesized that the prevalence estimate of these psychosocial disturbances would be significant and would increase throughout pregnancy. We further examined gestational age as a predictor of psychosocial outcomes over time.

## Methods

We conducted a longitudinal study among pregnant women in the Cape Coast, Ghana; details of the study have been described elsewhere [45]. In brief, women were recruited during their first trimester of pregnancy (< 13 weeks; *n* = 116) and followed through to their 2^nd^ (13–27 weeks; *n* = 71) and 3^rd^ (28–36 weeks; *n* = 71) trimesters. Multistage sampling was adopted to select seven antenatal health facilities in and around Cape-Coast, Ghana. Eligibility for this study included attendance at any of the seven selected prenatal clinics in Cape Coast, Ghana; aged between 18–38 years at enrolment; < 13 weeks gestation at enrolment (determined by last menstrual period or ultrasound scan); expecting a singleton pregnancy with no known congenital anomalies; and no known history of diabetes mellitus or hypertension. Questionnaires were used to collect data on anxiety symptoms, depressive symptoms and QoL at each trimester. Food insecurity was assessed using the eight items comprising the US Adult Food Security Scale on the 18-item US Household Food Security Survey Module [46, 47]. Trained enumerators interviewed participants across all 3 trimesters. Questionnaires were translated to Twi (common language spoken in Ghana) and Fante (local language in Cape Coast, Ghana), and back translated to English. After the first visit was completed, each participant was provided with a date for her 2^nd^ trimester visit. Participants were then followed into their 2^nd^ and 3^rd^ trimesters. At each health facility, a nurse was recruited to coordinate activities between patients and the enumerators. At the end of the first two visits, each woman received a bar of soap plus transportation cost as incentive, and at the end of the third visit, each woman received a baby onesie plus transportation cost.

### Assessment of psychosocial wellbeing

To ensure privacy and confidentiality, all psychosocial outcomes were assessed with an enumerator one-on-one either in a closed room in the health facility or in a quiet space. Depressive symptoms were assessed using the Center for Epidemiologic Studies Depression Scale [48] with reliability > 0.85 (CES-D), anxiety was assessed using the Beck Anxiety Inventory [49] with reliability of 0.92 (BAI), and QoL was assessed using the RAND 36-Item Short Form Health Survey [50] with reliability > 0.90 (RAND SF-36) at each trimester in pregnancy. The CES-D is a 20-item scale. Pregnant women were asked to rate their depressive symptoms on a scale of 0–3 for each item. Higher scores indicate higher depressive symptoms. Scores for items on the CES-D were summed with a cut off of ≥ 16 being indicative of elevated depressive symptoms [48]. Based on the psychometric analyses, the cut off for the CES-D scale was redefined for this population. Since a cut off ≥ 16 is indicative of depressive symptoms when the total possible score is 60, we divided the cut off by 60, (16/60) to obtain 0.2667. For this population, our psychometric analyses indicated that 13 items should be retained for a total possible score of 39 (13*3 = 39) and, therefore, we multiplied 0.2667 by 39 to obtain a cut off of ≥ 10 for this population.

The BAI is a 21-item self-reported questionnaire. Pregnant women were asked to rate their anxiety symptoms on a scale of 0–3 for each item. Higher scores indicate higher anxiety symptoms with the typical cut off point being ≥ 16 to indicate at least moderate anxiety. The same method applied for CES-D to obtain the population cut off was applied to the BAI to obtain a cut off of ≥ 14 based on the total number of items retained after psychometric analyses.

The RAND SF-36 measures 8 health constructs: physical functioning; role physical; bodily pain; social functioning; role emotional; general mental health (psychological distress and psychological wellbeing); vitality (energy/fatigue); and general health perceptions [51]. Total scores of 0–100 were obtained, with higher scores indicative of better QoL. A cut off of < 50 indicates low QoL, both before and after psychometric analyses.

### Statistical analyses

#### Exploratory factor analyses (EFA) for CES-D, BAI and RAND SF-36

The questionnaires used to assess our outcomes of interest were developed in Western countries and tests produced and standardized in one language or culture are not automatically valid in a setting that differs from the original population. Therefore, the psychometric properties were analyzed via factor analysis to determine the reliability of our measures.

Exploratory factor analysis using principal axis factoring with promax rotation was employed for each questionnaire based on 1^st^ trimester scores [52]. We used scree plot [53], parallel analysis [54] and minimum average partials [55] (MAP) to determine the number of factors to retain. Pattern coefficients ≥ 0.30 were considered salient on a factor and a minimum of three salient items were considered adequate on each factor [56]. Items with low salient loadings were deleted, and the reliability or internal consistency for each factor was examined using Cronbach’s alpha, with ≥ 0.70 considered adequate [57]. Other fit indices such as Akaike Information Criterion (AIC), Schwarz Information Criterion (SIC), Tucker–Lewis Index (TLI) and Root Mean Square Residual (RMSR) were examined to determine the best factor solution for all three scales (CES-D, BAI and RAND SF-36). The fit indices for the suggested factor solutions were compared. The smallest AIC and SIC were retained as well as TLI > 0.9 with RMSR < 0.05 [58, 59], as they indicate stronger evidence for the model. A meaningful percent variance explained by each factor was also used to determine the number of factors to retain. Factor solutions with adequate internal consistency, meaningful percent variance and theoretically meaningful patterns were selected for interpretation.

#### Descriptive statistics

Using the factor scores, we determined the proportion of pregnant women who had low psychosocial wellbeing (depressive and anxiety symptoms, and low QoL). For outcomes with multiple factors, a total factor score was calculated by first finding the product of the pattern coefficient and the raw scores to obtain a factor score for each item; summing these factor scores to obtain a factor total for each factor; then, the factor scores were summed to obtain a total factor score for each outcome. For example, the scores on the 4 factors for the BAI were added to obtain the total factor score for BAI. QoL on the other hand was calculated as the average of the factor scores since each score is calculated as a percentage (out of 100). The prevalence estimate for each outcome was reported using the published cut off as well as the population derived total factor cut off described above. Repeated measures ANOVA was used to determine changes in psychosocial outcomes over time. A Generalized Estimating Equation (GEE) model was used to determine if gestational age was a predictor of the psychosocial outcomes over time. GEE was chosen as it is a robust method that considers the longitudinal nature of our study, accounts for within-subject correlation and allows for a multivariable model.

## Results

### Factor scores

A one-factor solution with 13 items was obtained for the CES-D scale. TLI indicated good reliability (0.97) and small RMSR (0.05) indicated acceptable fit. The one-factor solution accounted for 100% of the variance with a Cronbach’s alpha of 0.84 and eigenvalue of 8.78. The pattern coefficients ranged from 0.30–0.84 with communality ranging from 0.09–0.70. The one-factor solution included items that describe depressive affect and interpersonal concerns with two positive affect items (Supplementary Table 1a).

A four-factor solution with 18 items was obtained for the BAI. Overall Cronbach’s alpha and TLI indicated good reliability (0.87) and small RMSR (0.05) indicated acceptable fit. The four-factor solution accounted for 98.9% of the cumulative variance. It had communality ranging from 0.21–0.67 and inter factor correlation between 0.27–0.47. Factor I had a Cronbach’s alpha of 0.84 and an eigenvalue of 16.22 and it explained 57.8% of the variance with pattern coefficients ranging from 0.41–0.92. It was named the “fear factor,” given the items included (Supplementary Table 1b). Factor II had a Cronbach’s alpha of 0.70 and an eigenvalue of 5.42 and it explained 19.3% of the variance with pattern coefficients ranging from 0.43–0.67. It was named the “nervous-factor,” given the items that loaded. Factor III had a Cronbach’s alpha of 0.76 and eigenvalue of 4.10 and it explained 14.6% of the variance with pattern coefficients ranging from 0.32–0.85. It was named the “panic factor.” Factor IV had a Cronbach’s alpha of 0.72 and eigenvalue of 2.34 and it explained 8.4% of the variance with pattern coefficients ranging from 0.31–0.95. Factor IV was named the “somatic factor.”

A four-factor solution with 26 items was obtained for the RAND SF-36. TLI and Cronbach’s alpha indicated good reliability (0.80 and 0.88, respectively) and small RMSR (0.078) indicated acceptable fit. The four-factor solution accounted for 100% of the cumulative variance with inter-factor correlations ranging from 0.16–0.50. Factor I had a Cronbach’s alpha of 0.86 and an eigenvalue of 14.39 and explained 52.4% of the variance, with pattern coefficients ranging from 0.44–0.78. We named it the “physical health factor”, given the items that loaded (Supplementary Table 1c). Factor II had a Cronbach’s alpha of 0.84 and an eigenvalue of 6.44 and it explained 23.5% of the variance, with pattern coefficients ranging from 0.37–0.89. It was named the “role physical factor”. Factor III had a Cronbach’s alpha of 0.79 and an eigenvalue of 3.62 and it explained 13.2% of the variance, with pattern coefficients ranging from 0.41–0.93. It was named the “role emotional factor” and finally, factor IV had a Cronbach’s alpha of 0.73 and eigenvalue of 3.02 and explained 11.0% of the variance, with pattern coefficients ranging from 0.30–0.79. Factor IV was named the “general health/vitality (GHV) factor”.

### Prevalence estimate of depressive symptoms, anxiety symptoms and low QoL

**Fig. 1:**
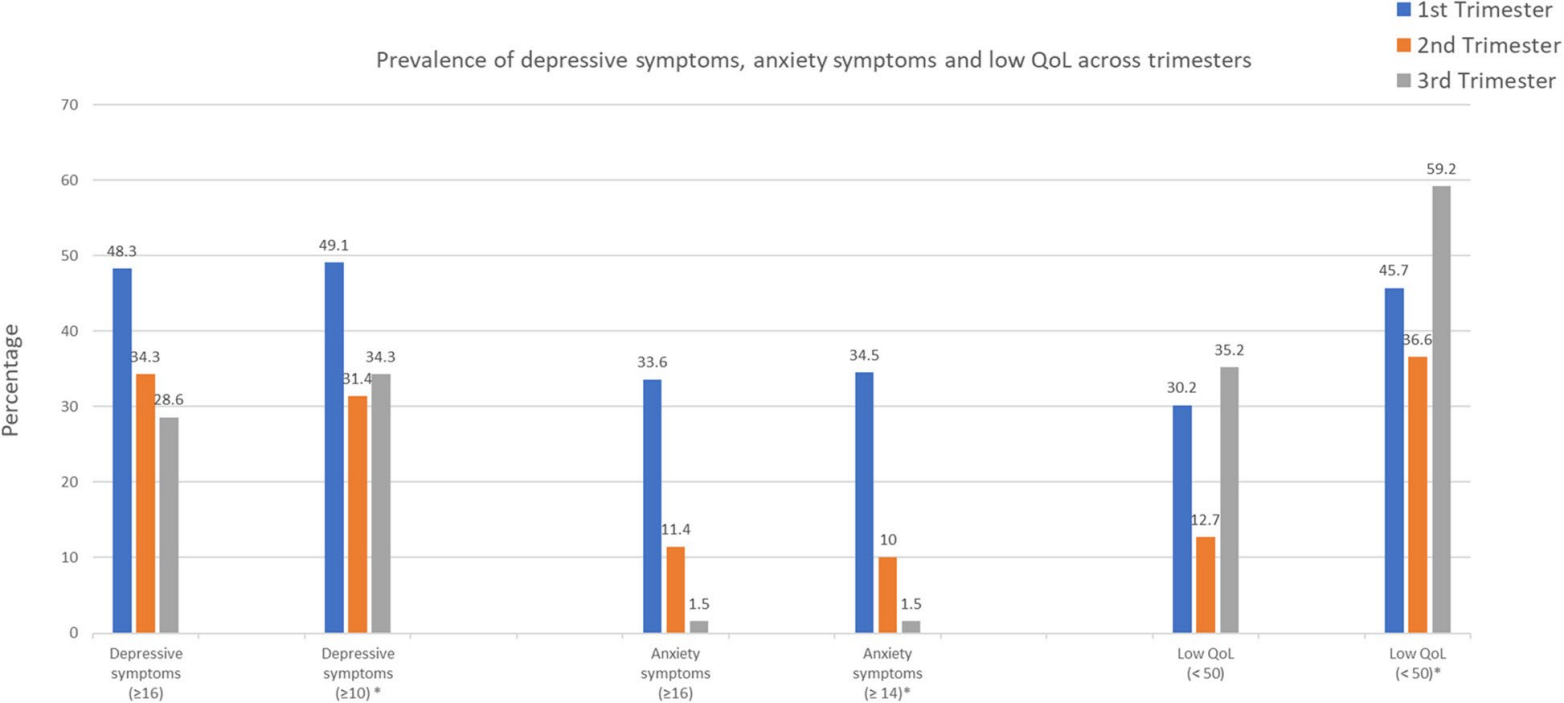
Prevalence estimate of depressive symptoms, anxiety symptoms and low QoL across trimesters. *Cut offs adjusted based on the results of the psychometric analyses

The prevalence estimate of depressive symptoms using the conventional cut off points (CESD ≥ 16) was 48%, 34%, 29%; anxiety symptoms (BAI ≥ 16) was 34%, 11%, 2% and low QoL (SF-36 < 50) was 30%, 13%, 35% for 1^st^, 2^nd^ and 3^rd^ trimesters, respectively. Cut offs based on number of items to retain from factor analyses, indicated 49%, 31% and 34% for depression (cut off ≥ 10); 35%, 10% and 2% (cut off ≥ 14) for BAI; and 46%, 37% and 59% for low QoL (cut off < 50) for 1^st^, 2^nd^ and 3^rd^ trimesters, respectively (Fig. 1).

### Change in depressive symptoms, anxiety symptoms and low QoL over time

Depressive symptoms decreased over time. Significantly higher depressive symptoms were found in the 1^st^ trimester (5.4 ± 0.3), compared with the 2^nd^ (4.2 ± 0.4) and 3^rd^ (4.0 ± 0.5) trimesters, with no significant differences between the 2^nd^ and 3^rd^ trimester scores (Table 1). Gestational age was a significant predictor of depressive symptoms; a one-week increase in gestational age decreased depressive symptoms by 0.073 units (Table 2). After controlling for sociodemographic variables such as parity, marital status and food insecurity, a one week increase in gestational age significantly decreased depressive symptoms by 0.048 units. Parity and food insecurity contributed significantly to predicting depressive symptoms.

**Table 1:** Repeated measures ANOVA for change in depressive symptoms, anxiety symptoms and QoL over time*

Dependent Variable		F-value	*p*-value (change over time)	Trimester 1 (mean ± SE)	Trimester 2 (mean ± SE)	Trimester 3 (mean ± SE)
Factor scores	**Depressive symptoms**	4.22	0.0158	5.40 ± 0.34^a^	4.21 ± 0.44^b^	4.00 ± 0.45^b^
	**Total Anxiety symptoms**	20.83	< 0.0001	5.66 ± 0.43^a^	2.65 ± 0.54^b^	2.00 ± 0.54^b^
	Fear Factor	8.39	0.0003	1.56 ± 0.17^a^	0.84 ± 0.22^b^	0.56 ± 0.22^b^
	Nervous Factor	16.68	< 0.0001	1.69 ± 0.14^a^	0.61 ± 0.17^b^	0.88 ± 0.18^b^
	Panic Factor	8.53	0.0003	1.07 ± 0.12^a^	0.63 ± 0.16^a,b^	0.25 ± 0.16^b^
	Somatic Factor	20.43	< 0.0001	2.32 ± 0.69^a^	1.56 ± 0.70^b^	1.32 ± 0.71^b^
	**Total QoL**	9.85	< 0.0001	34.24 ± 1.24^a^	36.05 ± 1.58^a^	27.25 ± 1.59^b^
	Physical Health	22.14	< 0.0001	48.04 ± 1.45^a^	44.15 ± 1.45^a^	35.91 ± 1.46^b^
	Role Physical	6.21	0.0023	28.25 ± 2.34^a^	29.25 ± 2.98^a^	16.74 ± 2.99^b^
	Role Emotional	4.18	0.0163	40.65 ± 2.54^a,b^	43.89 ± 3.22^a^	31.89 ± 3.24^b^
	GHV	24.46	< 0.0001	19.74 ± 0.72^a^	26.95 ± 0.91^b^	24.46 ± 0.92^b^

*Means with different letter superscripts within a row are significantly different. *QoL* quality of life, *GHV* general health/vitality

**Table 2:** Predictors of psychosocial outcomes based on gestational and sociodemographic variables

Dependent Variable		Predictor	Parameter Estimate	95% CI	*p*-value
Depressive symptoms	Univariate	Gestational age	*-*0.073	-0.115, -0.031	0.001
	Multivariate	Gestational age	-0.048	-0.085, -0.011	0.012
		Parity	0.412	0.103, 0.721	0.009
		Marital status	0.816	-0.089, 1.720	0.077
		Food insecurity	0.594	0.428,0.761	< 0.001
Total Anxiety symptoms	Univariate	Gestational age	-0.187	-0.235, -0.139	< 0.001
	Multivariate	Gestational age	-0.158	-0.205, -0.111	< 0.001
		Parity	0.162	-0.277, 0.601	0.470
		Marital status	0.145	-0.950, 1.239	0.796
		Food insecurity	0.619	0.389, 0.849	< 0.001
Total QoL	Univariate	Gestational age	-0.187	-0.347, -0.027	0.028
	Multivariate	Gestational age	-0.247	-0.398, -0.097	0.001
		Parity	0.491	-0.794, 1.776	0.454
		Marital status	1.607	-2.140, 5.354	0.401
		Food insecurity	-1.385	-1.983, -0.787	< 0.001

Generalized Estimating Equation models with univariate and multivariate models

Similarly, anxiety symptoms decreased over time. A significant difference was found on the total factor score for anxiety as well as each individual factor score between the 1^st^ and 2^nd^ and the 1^st^ and 3^rd^ trimesters but not between the 2^nd^ and 3^rd^ trimesters, except for the panic factor (significant difference only between the 1^st^ and 3^rd^ trimesters). Gestational age was a predictor of anxiety symptoms even after controlling for parity, marital status and food insecurity; a one-week increase in gestational age was associated with a decrease in the total factor score for anxiety by 0.158. Food insecurity was the only sociodemographic variable that significantly predicted total anxiety scores.

There was a slight difference in the pattern of change over time for QoL as compared with depression and anxiety. For the total factor QoL score, there were no significant differences between 1^st^ and 2^nd^ trimesters; however, women in their 1^st^ and 2^nd^ trimesters had significantly higher QoL than those in their 3^rd^ trimester. Scores on the role emotional and GHV factors showed a different trend. Role emotional scores were lowest in the 3^rd^ trimester, with significantly higher scores in the 2^nd^ but not the 1^st^ trimester. Scores did not differ between 1^st^ and 2^nd^ or 1^st^ and 3^rd^ trimester. GHV scores were significantly lower in the 1^st^ than the 2^nd^ and 3^rd^ trimesters, but not different between the 2^nd^ and 3^rd^ trimesters. Gestational age significantly predicted total QoL score even after controlling for parity, marital status and food insecurity; a one-week increase in gestational age was associated with a decrease in total QoL by 0.247 units, (Table 2). Food insecurity was the only sociodemographic variable that significantly predicted QoL.

## Discussion

### Depressive symptoms during pregnancy

In line with our hypothesis, depressive symptoms were found to be highly prevalent throughout pregnancy in Cape Coast, Ghana; however, counter to our hypothesis, this prevalence estimate decreased during the course of pregnancy. Even so, the high prevalence of depressive symptoms in this population was of public health significance during all trimesters of pregnancy. In Ghana, like many developing countries, depressive symptoms are not usually assessed during pregnancy due to low priority of mental health, lack of mental health facilities, insufficient routine data collection on mental health and lack of mental health data for planning [44]. Additional cultural reasons for this neglect include the stigmatization of depression, leading women who are depressed to not seek psychiatric treatment due to fear of being labeled with psychosis [3]. Instead, many opt to seek treatment and counselling from traditional and religious healers, due to cultural acceptance [60]. As such, the magnitude of the problem is not realized and diagnosed in clinical settings. We have shown that a high prevalence estimate of depressive symptoms exist during pregnancy in this population, indicating that policies should be put in place to prioritize the assessment of depressive symptoms during pregnancy to avoid or diminish its effects on mothers and their fetus.

The prevalence estimates of depressive symptoms in our study are much higher than rates found in most studies conducted in developed countries. Schmied et al. [30] observed depressive symptom rates of 8.7% in Australia and New Zealand while Underwood et al. [61] observed 17% prevalence of antenatal depression across the entire pregnancy from a review involving twelve developed countries. Bennett et al. [16] observed rates of 7.4%, 12.8% and 12% in the 1^st^, 2^nd^ and 3^rd^ trimesters, respectively, in a review covering 21 developed countries. In Europe, prevalence rates of 12% and 14% were found in the 2^nd^ and 3^rd^ trimesters of pregnancy, respectively [62]. A study in rural US conducted during pregnancy observed rates similar to ours, 33% [63]. In developing countries, most reported rates are higher but still not as high as what we found. A review by Gelaye et al. [31] in low-income countries observed a pooled prevalence of 25%. Previous studies in Ghana found prevalence rates of 26% in the 3^rd^ trimester of pregnancy [41, 42] a rate comparable to what we found in our 3^rd^ trimester (29%) using the Western cut off (≥ 16). Our factor proportional cut off (≥ 14) however, gave a higher prevalence estimate of 34% in the 3^rd^ trimester. In the few studies that have measured depressive symptoms longitudinally during pregnancy, some reported increasing rates [16, 30], but we found decreasing rates in our population. The previous longitudinal studies were conducted in developed countries and, even though the findings indicate increasing rates throughout pregnancy, the rates reported in the 3^rd^ trimester are still not as high as rates reported during each of the trimesters in this study. While the use of different instruments and cutoffs might explain some of the differences, (most studies have used the Beck Depression Inventory (BDI) or the Edinburgh Postnatal Depression Scale (EPDS), while we used the CES-D), there is the possibility that the construct of depression is conceptualized differently in Ghana and there may be the need for better instruments that truly capture depressive symptoms in this setting. Despite the high prevalence of depressive symptoms observed in this population, depressive symptoms decreased with gestational age even after controlling for sociodemographic characteristics. Similar to previous findings, parity and food insecurity were significant predictors of depressive symptoms [19, 22]. This finding emphasizes the importance of addressing the issue of food insecurity not only to solve the physical needs of the pregnant woman but also the psychological needs.

### Anxiety during pregnancy

Our findings suggest that estimated prevalence of anxiety symptoms is high in the 1^st^ trimester but low in the 2^nd^ and 3^rd^ trimesters of pregnancy. A review by Schmied et al. [30] observed anxiety symptom rates of between 8 to 10% during the entire pregnancy. Our rates are much higher in the 1^st^ trimester (34%) than rates found in most studies. Despite this, our prevalence rates during the 2^nd^ and 3^rd^ trimesters are comparable to other studies. A study in Nigeria found the prevalence of anxiety symptoms to be 13%, 4% and 6% in the 1^st^, 2^nd^ and 3^rd^ trimesters, respectively [17]. A study in Kumasi, Ghana, observed a higher prevalence rate of 11% in the 3^rd^ trimester, compared to what we observed [41]. One reason for the discrepant findings could be the different instruments used to assess anxiety symptoms; our study used the BAI while the study in Kumasi used the 7-item Anxiety Scale (GAD-7).

We were surprised by the finding of a high estimated prevalence of anxiety in the 1^st^ trimester but not the 2^nd^ or 3^rd^ trimesters. When we considered gestational age, we found total anxiety, fear, nervous, panic and somatic symptoms decreased with increasing gestational age. There may be cultural reasons that explain the prevalence of anxiety in this population. One might be the fear of pregnancy, especially during the first trimester. In Ghanaian settings, most women are first informed of their pregnancy status when they visit the clinic. For instance, a woman may present with symptoms that resemble malaria, and may have been treated for malaria over a period of time but the symptoms did not improve. She may then report to the clinic, only to be told that she is pregnant. Thus, the news of her pregnancy may come as a surprise. This may cause a woman to be anxious, especially during the first trimester [64–66]. Additionally, a woman might be concerned about her husband/partner accepting a new baby [67]. If the woman is not married, it poses multiple challenges including who this baby belongs to, whether the man responsible will accept it or not, and how the community will handle her pregnancy since being pregnant outside of marriage is frowned upon [68, 69]. Another cultural reason that may add to a woman’s anxiety is the fact that a woman in her first trimester will tend to hide her pregnancy and not share her news until she is visibly pregnant. This is due to the belief that if people get to know of her pregnancy she might be “bewitched” or “something bad will happen” and she might lose her baby [70]. This belief is borne out of the high rates of miscarriage observed during the first trimester of pregnancy [71]. Food insecurity may also be an issue. If a woman already has a child/children and she is not prepared for another pregnancy, this may be a cause of worry. In our study, we found the prevalence of food insecurity to be 50%, 30% and 25% for the 1^st^, 2^nd^ and 3^rd^ trimesters, respectively and food insecurity and gestational age were significant predictors of anxiety symptoms; one unit increase in food insecurity increased total anxiety symptoms by 0.619 units. Previous research indicates that food insecurity is an issue in this population and this can be a cause of anxiety and depression [72, 73]. As far as changes in the prevalence of anxiety over the course of pregnancy, once the news of the pregnancy is announced, if the husbands/partners and family members are happy with the pregnancy and are in support of the woman being pregnant, the woman’s worry, fear and panic may decrease, thus reducing anxiety as the pregnancy advances. This may account for the decreased prevalence estimate of anxiety symptoms seen during the 2^nd^ trimester. By the 3^rd^ trimester, generally, the Ghanaian family and society is happy to receive a new baby and this may lead even anxious women to become less anxious by the end of the pregnancy [16]. The high dropout rate observed between the 1^st^ and 2^nd^ trimesters could also account for a decrease in anxiety symptoms, assuming women who were anxious were those who dropped out. However, there were no significant differences in anxiety symptoms between women who dropped out and those who did not.

The instruments used to assess psychosocial health could also account for the low estimated prevalence of anxiety yet high depressive symptoms observed in the population. The BAI and CES-D, even though widely used by clinicians and researchers to determine anxiety and depressive symptoms, respectively, may not be as appropriate among pregnant women in the Ghanaian culture as they are in Western cultures. For instance, items may be interpreted differently among Ghanaian women than Western women. Even though the psychometrics were run to determine cultural appropriateness, we may have missed certain constructs that may describe anxiety or depressive symptoms in this population as factors that may determine anxiety and depression in the Ghanaian culture may be different from factors in Western populations. This research highlights the importance of developing valid cultural psychosocial measures that consider and understand how people from different cultures think about mental health and mental health problems. Research by De-Graft Aikins and Ofori-Atta [74] found symptoms of mental illness in Ghana to be characterized by excessive thinking, worry, persistent physical symptoms such as headaches, bodily pain, stresses arising from multiple responsibilities from family and work, and financial hardship. These symptoms are not listed on either the BAI or CES-D scales. It may be important to capture some of these factors in assessing anxiety or depression in the Ghanaian population.

### QoL during pregnancy

We hypothesized that a significant number of the women would have low QoL and that this estimated prevalence would increase over time. QoL did change significantly over time with women in the 3^rd^ trimester having a significantly lower mean QoL, lower physical health and lower role physical scores compared with the 1^st^ and 2^nd^ trimesters. Our study also found that GHV scores were lowest in pregnant women in their 1^st^ trimester, and scores did not differ significantly between the 2^nd^ and 3^rd^ trimesters. Our results are similar to those of Chang et al. who found that pregnant Taiwanese women increased in GHV across trimesters with a significant difference between early and mid-pregnancy but no difference between mid and late pregnancy [33]. We found that pregnant women in their 3^rd^ trimester had the lowest role emotional scores, but this did not differ from those during their 1^st^ trimester. Our results agree with findings from two earlier studies which found that role physical scores decreased from early to late pregnancy but there was no difference between early to mid-pregnancy role physical scores [32, 33]. Chang et al*.* also found that role emotional scores were stable throughout pregnancy, which was similar to our results. Our findings may be related to common symptoms in early pregnancy such as feeling weak, low energy, nausea and vomiting [75] which are unwanted side effects experienced as a result of the hormonal changes that occur. These symptoms not only affect the physical health of pregnant women but can also negatively impact their psychological function [37]. In the 2^nd^ and 3^rd^ trimesters however, these symptoms may disappear, and women may gain more energy, thus improving GHV.

We found that gestational age was a negative predictor for total QoL, even after controlling for parity, marital status and food insecurity. Our findings agree with a study in France by Morin et al. [34], who assessed QoL at each month during pregnancy and found that QoL decreased significantly over time during pregnancy and decreased further between the 4^th^ and 8^th^ months. Similar to our study, Hueston and Kasik-Miller also found that physical health and role physical domains decreased with gestational age [32]. It is important to note that food insecurity was a significant predictor of total QoL and this emphasizes the need to address food insecurity particularly among pregnant women to improve QoL during pregnancy.

The main limitation of this study was sample size due to a dropout rate of 37.8% from the 1^st^ to 2^nd^ trimesters. Reasons such as miscarriages, husband refusing participation of their spouse in research, unanswered phone calls, phone switched off, and relocation accounted for the high dropout rate in this study. Another limitation could potentially be the new cut off developed for this population which must be validated in other studies, even though the prevalence estimate of psychosocial outcomes between the two cut offs was not different. Strengths of this study include the longitudinal nature which assessed psychosocial outcomes throughout pregnancy and including a population and culture which has been understudied. Another strength is the fact that we ran psychometric analyses on the psychosocial outcome scales and thus, we are confident of the results of our analyses.

## Conclusion

Our findings suggest that the estimated prevalence of depressive symptoms and low QoL are high in the 1^st^, 2^nd^ and 3^rd^ trimesters of pregnancy in Cape Coast, Ghana, whereas prevalence estimate of anxiety symptoms are high in the 1^st^ trimester only. It is imperative that measures be put in place to encourage policy makers to include screening for such disturbances during regular antenatal care. This calls for education among the populace, as well as more research and prioritization of existing resources to argue for greater attention to mental health in general, especially anxiety, depressive symptoms, and quality of life and their evaluation during antenatal care. The issue of food insecurity as a contributing factor to the psychosocial issues observed cannot be ignored. Food insecurity must be given immediate attention to help cater to the physical demands of pregnant women and also address their psychological needs.

## Supplementary Information

**Additional file 1:** **Supplementary Table 1a:** Pattern coefficients from EFA showing the one-factor solution for CES-D. **Supplementary Table 1b:** Pattern coefficients from EFA showing the four-factor solution for the BAI. **Supplementary Table 1c:** Pattern coefficients from EFA showing the four-factor solution for the RAND SF-36.

## Data Availability

The datasets generated and/or analysed during the current study are available from the corresponding author on reasonable request.

## Abbreviations

QoL: Quality of life
CES-D: Center for Epidemiological Studies Depression Inventory
BAI: Beck Anxiety Inventory
AIC: Akaike Information Criterion
SIC: Schwarz Information Criterion
TLI: Tucker–Lewis Index
RMSR: Root Mean Square Residual
ANOVA: Analysis of Variance
GHV: General Health/Vitality
