# Supplementary material for: High levels of depressive symptoms and low quality of life are reported during pregnancy in Cape Coast, Ghana; a longitudinal study
Source: BMC Public Health. 2022 May 5;22:894. doi: 10.1186/s12889-022-13299-2 (PMC9069749; doi:10.1186/s12889-022-13299-2)
Supplement: Supplementary file 1 — Additional file 1: Supplementary Table 1a: Pattern coefficients from EFA showing the one-factor solution for CES-D. Supplementary Table 1b: Pattern coefficients from EFA showing the four-factor solution for the BAI. Supplementary Table 1c: Pattern coefficients from EFA showing the four-factor solution for the RAND SF-36. [file 12889_2022_13299_MOESM1_ESM.docx]

High levels of depressive symptoms and low quality of life are reported during pregnancy in Cape Coast, Ghana; a longitudinal study

Ruth Adisetu Pobee^1^, Jacob Setorglo^2^, Moses Kwashie Klevor^2^, Laura E. Murray-Kolb^1,3*^

^1^Department of Nutritional Sciences, The Pennsylvania State University, USA; ^2^Department of Clinical Nutrition and Dietetics, University of Cape Coast, Ghana; ^3^Department of Nutrition Science, Purdue University, USA

*Corresponding Author: Laura E. Murray-Kolb, Room 214 Stone Hall, 700 West State Street, West Lafayette, IN 47907, lmurrayk@purdue.edu

**Supplementary Table 1a**

Pattern coefficients from EFA showing the one-factor solution for CES-D

|  | Questions | Factor 1 | h ^2^ |
| --- | --- | --- | --- |
| 1.  2.  3.  4.  5.  6.  7.  8.  9.  10.  11.  12.  13. | I was bothered by things that usually don't **bother** me  I felt that I could not shake off the **blues** even with help from my family or friends  I had trouble keeping my **mind** on what I was doing  I felt **depressed**  I felt that everything I did was an **effort**  My sleep was **restless**  I was **happy**  I felt **lonely**  People were **unfriendly**  I **enjoy**ed life  I had **cry**ing spells  I felt **sad**  I felt that people **disliked** me  Eigenvalue  % Variance  Cronbach’s Alpha | 0.37765  0.80422  0.49463  0.79545  0.40622  0.30081  0.39331  0.39170  0.52817  0.41586  0.61396  0.83878  0.57841  8.78  100.00  0.84 | 0.14262008  0.64676619  0.24466112  0.63273477  0.16501738  0.09048778  0.15469552  0.15343187  0.27896729  0.17293720  0.37694779  0.70355171  0.33456261 |

**Supplementary Table 1b**

Pattern coefficients from EFA showing the four-factor solution for the BAI

| Items | Fear factor | Nervous factor | Panic factor | Somatic factor | h ^2^ |
| --- | --- | --- | --- | --- | --- |
| Scared  Fear of dying  Terrified/afraid  Fear of worse  Numbness/tingling  Hot/cold sweat  Wobbliness in legs  Feeling hot  Unable to relax  Face flushed  Unsteady  Shaky  Feeling of choking  Fear of losing control  Faint/lightheaded  Dizzy  Heart pounding/racing  Hands trembling  Eigenvalue  % Variance  Cronbach’s Alpha | **0.91860**  **0.75759**  **0.73307**  **0.40581**  -0.04495  -0.11865  0.13108  0.00513  -0.04695  0.11152  0.06541  -0.07140  0.16862  0.26188  -0.02431  0.19477  0.19055  0.07253  16.22  57.75  0.84 | -0.05512  -0.07894  0.13181  0.17670  **0.67349**  **0.58867**  **0.58815**  **0.50838**  **0.48867**  **0.42548**  0.04130  -0.07263  0.28602  -0.03288  -0.06662  -0.00695  0.21522  0.22359      5.42  19.30  0.70 | -0.02245  0.13652  -0.02897  -0.01986  -0.00181  0.15525  -0.01143  -0.08616  0.13198  -0.03787  **0.84699**  **0.83225**  **0.33007**  **0.32020**  0.03799  0.20873  0.14002  -0.19805    4.10  14.61  0.76 | 0.07278  -0.08226  0.08673  0.15710  -0.12218  0.19985  -0.17028  0.23361  0.17839  0.20796  -0.01931  0.15206  -0.23699  0.00293  **0.94751**  **0.41850**  **0.34054**  **0.31263**    2.34  8.34  0.72 | 0.38982793  0.36536329  0.37827939  0.36975447  0.34265259  0.39696646  0.41905527  0.76886810  0.68883478  0.28111524  0.25721102  0.73190494  0.21380282  0.56325950  0.83525713  0.87004953  0.34172219  0.48198731 |

**Supplementary Table 1c**

Pattern coefficients from EFA showing the four-factor solution for the RAND SF-36

| Items | Physical Health | Role  Physical | Role Emotional | General Health and vitality | h ^2^ |
| --- | --- | --- | --- | --- | --- |
| Does your health now limits you in these activities  Climb one stair  Lifting  Climb several stairs  Moderate activity  Walk several blocks  Bending  Walk one block  Bath/dress up  Walk a mile  Vigorous activity  Have you had the following problems with your work?  Accomplished less  Cut time on work  Difficult to work  Limited in work  Physical/emotions interfering with social life  Do emotional problems interfere with the following?  Cut down on time spent working  Accomplish less than you would  Do work as careful as usual  Have you felt so down in dumps?  Have you felt  Lot of energy  Full of Pep  Excellent health  Worn-out  Tired  Down hearted and blue  As healthy as anyone  Eigenvalue  %variance  Cronbach’s Alpha | **0.78426**  **0.74079**  **0.73161**  **0.67170**  **0.65348**  **0.55557**  **0.54117**  **0.51358**  **0.51129**  **0.44258**  -0.04733  -0.03123  -0.00336  0.06576  0.26032  -0.02100  -0.00250  0.04141  -0.15068  -0.09959  -0.04121  0.05039  0.17285  0.07484  -0.14204  0.21092  14.39  52.38  0.86 | -0.28776  -0.04239  -0.02691  0.05914  0.14476  0.15521  0.02489  -0.01796  0.09229  0.09448  **0.88990**  **0.82710**  **0.76635**  **0.65402**  **0.37496**  -0.04437  -0.04736  0.10954  0.15065  -0.09472  -0.18613  0.15777  0.20440  0.24512  0.23420  0.17800  6.44  23.45  0.84 | 0.08934  -0.03280  0.02699  -0.00906  -0.20949  0.08642  -0.10421  0.04564  -0.11209  0.18696  0.02211  -0.07463  -0.00225  0.09531  0.05424  **0.92579**  **0.85987**  **0.56983**  **0.41046**  -0.17065  -0.04570  0.14546  0.14556  0.10430  0.23041  0.12624  3.62  13.17  0.79 | 0.01878  -0.10572  0.08468  -0.03644  -0.02669  -0.09816  -0.03344  -0.10285  0.21316  0.08652  -0.10647  -0.19832  -0.00933  0.12342  0.11907  -0.11170  -0.02766  -0.05900  0.18640    **0.79495**  **0.61879**  **0.43636**  **0.37106**  **0.36606**  **0.31341**  **0.30359**  3.02  11.00  0.73 | 0.53537672  0.49361066  0.56625339  0.47114399  0.47224325  0.41253974  0.28205072  0.25115179  0.38958683  0.36085223  0.74093838  0.5657511  0.57969332  0.60786049  0.37133472  0.79428777  0.69408510  0.40163238  0.29320388  0.58493847  0.35069817  0.34101074  0.39032136  0.42542293  0.28212826  0.32193360 |
